# Eight brain structures mediate the age-related alterations of the working memory: forward and backward digit span tasks

**DOI:** 10.3389/fpsyg.2024.1377342

**Published:** 2024-09-03

**Authors:** Maryam Bahri, Hassan Farrahi, Hami Mahdavinataj, Seyed Amir Hossein Batouli

**Affiliations:** ^1^Department of Neuroscience and Addiction Studies, School of Advanced Technologies in Medicine, Tehran University of Medical Sciences, Tehran, Iran; ^2^Kavosh Cognitive Behavior Sciences and Addiction Research Center, Department of Psychiatry, School of Medicine, Guilan University of Medical Sciences, Rasht, Iran; ^3^BrainEE Research Group, Tehran University of Medical Sciences, Tehran, Iran

**Keywords:** working memory, aging, MRI, resting-state network, voxel-based morphometry

## Abstract

**Introduction:**

Working memory (WM) as one of the executive functions is an essential neurocognitive ability for daily life. Findings have suggested that aging is often associated with working memory and neural decline, but the brain structures and resting-state brain networks that mediate age-related differences in WM remain unclear.

**Methods:**

A sample consisting of 252 healthy participants in the age range of 20 to 70years was used. Several cognitive tasks, including the n-back task and the forward and backward digit span tests were used. Also, resting-state functional imaging, as well as structural imaging using a 3T MRI scanner, were performed, resulting in 85 gray matter volumes and five resting-state networks, namely the anterior and posterior default mode, the right and left executive control, and the salience networks. Also, mediation analyses were used to investigate the role of gray matter volumes and resting-state networks in the relationship between age and WM.

**Results:**

Behaviorally, aging was associated with decreased performance in the digit span task. Also, aging was associated with a decreased gray matter volume in 80 brain regions, and with a decreased activity in the anterior default mode network, executive control, and salience networks. Importantly, the path analysis showed that the GMV of the medial orbitofrontal, precentral, parieto-occipital, amygdala, middle occipital, posterior cingulate, and thalamus areas mediated the age-related differences in the forward digit span task, and the GMV of superior temporal gyrus mediated the age-related differences in the backward digit span task.

**Discussion:**

This study identified the brain structures mediating the relationship between age and working memory, and we hope that our research provides an opportunity for early detection of individuals at risk of age-related memory decline.

## 1 Introduction

Normal aging begins a series of gradual changes in the human brain (Batouli et al., 2014b). In particular, cognitive neuroscientists have reported that regardless of the conditions of dementia or mild cognitive impairment (MCI), aging is associated with a decline in a set of essential cognitive functions, especially working memory (WM) performance (Bosnes et al., 2022; Sisakhti et al., 2024; Klencklen et al., 2017; Sisakhti et al., 2023). Working memory is considered an executive function skill that has a limited capacity to store and manipulate information temporarily. To put it more clearly, working memory is considered a vital brain system that provides the storage and manipulation of information required for other complex cognitive tasks such as reasoning, comprehension, learning, and language (Baddeley, 1992).

It has been reported that the degree of decline in cognitive abilities varies among older adults (Wilson et al., 2002). This means that a mild decrease in cognitive functions is observed in some older adults, while in others, a significant change in cognitive functions is observed (Cohen et al., 2019). These observed individual differences in older adults suggest that other factors mediate the age-cognition relationship. Despite existing scientific reports on age-related decline in working memory (Fabiani, 2012), several factors positively or negatively mediate the relationship between age and working memory that may increase or decrease this decline (Cansino et al., 2018). Given that brain changes are common in older age (Lockhart and DeCarli, 2014) and may affect cognition, various brain measures can be considered as mediators to clarify the relationship between age and working memory.

Among the factors that can be considered as a mediator between age and working memory is the brain structure. Examinations of brain volume in elderly adults suggest that the gray matter volume (GMV) and white matter (WM) volumes of the brain decrease with age (Driscoll et al., 2009; Farokhian et al., 2017; Giorgio et al., 2010). For example, GMV has been estimated to decrease by about 3 to 5% per decade (Resnick et al., 2003; Sisakhti et al., 2022). It is noteworthy that the degree of brain atrophy, like cognitive abilities, is not a homogeneous process throughout the brain (Fjell et al., 2014). The frontal and temporal lobes, which are involved in cognitive functions, show the greatest age-related decline in GMV (Alexander et al., 2006). On the other hand, studies have reported the relationship between the reduction of cognitive functions and the atrophy of brain areas involved in these abilities (Leong et al., 2017; Lövdén et al., 2013; Ramanoël et al., 2018). Brain atrophy refers to the loss of brain cells (neurons) and the connections between them, which can lead to a decrease in brain volume (Fjell et al., 2009). Brain atrophy in aging refers to the gradual loss of brain tissue and volume that occurs naturally as people age. This process is characterized by several morphological changes, including cortical thinning, white and gray matter volume loss, ventricular enlargement, and loss of gyri (Double et al., 1996).

Another important factor that is considered as a mediator between age and working memory is the resting-state brain networks (RSNs). Functional brain networks that exhibit synchronized activity during periods of rest—when an individual is not actively engaged in a specific task—are referred to as resting-state networks (Rosazza and Minati, 2011). Humans spend a significant portion of their day, estimated to be up to 50%, in mental states where their brain is not actively engaged in a specific task or cognitive activity. During these periods, the brain is essentially resting or operating in a task-free condition (Lurie et al., 2020). The patterns of resting-state functional connectivity resemble the patterns of activation seen during cognitive tasks, with up to 80% of their variation being similar (Cole et al., 2014, 2016). Evidence suggests age-related changes in the resting-state networks (Huang et al., 2015; Jockwitz et al., 2017), and almost every cognitive network has been shown to experience some degree of age-related decline (Varangis et al., 2019). This age-related decline at the network level includes a decrease in local efficiency at the network level (Song et al., 2014) and a decrease in connectivity within the network (Geerligs et al., 2015).

There are reports on the relationships between age and declined working memory (Cansino et al., 2013), between age and brain structure and resting state networks (Kaup et al., 2011; Varangis et al., 2019), and between brain structure and resting state networks and working memory (MacHizawa et al., 2020; Osaka et al., 2021). For example, a study conducted by Cansino et al. (2013) demonstrated that as individuals age, their verbal and visuospatial working memory abilities decline. This decrease is more closely linked to the cognitive resources required by the task rather than the nature of the information being processed (verbal or visuospatial). On the other hand, Kaup et al. (2011) conducted a review study indicating a positive correlation between the size of the hippocampal formation and memory performance in elderly individuals. Also, Varangis et al. (2019) demonstrated a deleterious effect of age on segregation and local efficiency and within-network connectivity of resting state networks in the brain. In addition, MacHizawa et al. (2020) reported a positive relationship between gray matter volume and visual working memory, in such a way that gray matter volume in the left lateral occipital region and right parietal lobe relates to the capacity and precision of visual working memory, respectively. In another study, Osaka et al. (2021) investigated the connectivity of resting-state networks in individuals with high and low working memory capacity. The results indicated a strong connection between dorsal attention and salience networks in individuals with high working memory capacity.

According to this knowledge, it can be hypothesized that the changes in the brain due to aging are responsible for the changes in working memory that occur with age. At first glance, this conclusion may appear to be correct, but simple correlations do not allow one to prove causality. Although there are several studies examining the neural correlates of age-related changes in working memory (Archer et al., 2018; Mattay et al., 2006; Rypma and D’Esposito, 2000; Schulze et al., 2011), to the best of our knowledge, previous studies were not based on testing a mediation model. The evidence for which brain structures are the neural substrates of age-related working memory decline is weak. In general, the search for the exact neural bases for working memory in normal aging has brought diverse results. Among the factors that led studies to achieve diverse results are the use of small samples or samples with a narrow age range, and the use of different cognitive tasks. Although there are mediation studies that examine the age-related difference in the tasks measuring WM (Bender and Raz, 2012; Cansino et al., 2018; Van Gerven et al., 2007; Zuber et al., 2019), to the best of our knowledge, none of these previous studies investigated the mediating role of gray matter volume and resting-state networks. For example, previous studies have examined the mediating role of factors such as inhibition (Van Gerven et al., 2007), executive functions (i.e., updating, inhibition, and shifting; Zuber et al., 2019), physiological traits, and individual characteristics (such as cultural and social activities; Cansino et al., 2018) in the relationship between age and working memory. Therefore, it has not yet been explicitly tested which brain structures and resting-state brain networks mediate the age-related decline in working memory.

Given that neurological changes can occur before the onset of cognitive decline (Coupé et al., 2019), identifying the brain structures and resting-state brain networks which mediate the relationship between age and working memory may provide an opportunity to slow or prevent memory loss in the elderly. The present study aims, therefore, to investigate which brain structures and resting-state brain networks mediate age-related differences in working memory.

## 2 Methods

### 2.1 Participants and procedure

We recruited 252 participants from the Iranian brain imaging database (*IBID*; Batouli et al., 2021). The inclusion criteria in the study were that the participants should be aged from 20 to 70 years, have completed at least 12 years of education, should be able to read, and have consent to participate in all stages of the research, in accordance with previous works (Batouli and Sisakhti, 2020). By selecting a wider age range, the study can capture a more comprehensive understanding of the trends and patterns in the population. This approach allows for the examination of age-related effects across a spectrum of adulthood rather than focusing solely on the extremes of age. Also, by not strictly dividing the participants into young and old groups, we aimed to avoid confounding factors that could arise from comparing two distinct age groups. This could lead to more nuanced findings that reflect gradual changes in health rather than sudden differences attributable to age alone. Also, they were Iranian, and Persian was their first or second language. The exclusion criteria were as follows: neurological or severe somatic disorder, pregnancy or breastfeeding, weight above 110 kg, previous use of drugs for neurological disorders, long-term history of drug use (except aspirin, vitamins, antibiotics, pain relievers, sleeping pills, anti-nausea drugs, and vaccinations), drug use or alcohol addiction (only based on the subjective report), and MRI contraindications.

The participants were distributed in 5 age groups: 59 participants in the early adult group (20–30 years old, 30 females), 62 participants in the early middle-aged adult group (30–40 years old, 31 females), 55 participants in the late middle-aged adult group (40–50, 31 females), 50 participants in the late adult’s group (50–60, 27 females), and 26 participants in the older adult group (60–70, 14 females). In this study, to achieve the goal of the research, we used several cognitive tasks, including the n-back task and the forward and backward digit span tests. Also, several Magnetic Resonance Imaging (MRI) protocols were performed. The procedures of data collection in the *IBID* study have been extensively documented in previous reports (Batouli et al., 2021). See Table 1 for demographic information of the participants by their age group. The ethical approval code for this study was IR.NIMAD.REC.1396.319, issued by the National Institute for Medical Research Development, in agreement with the Declaration of Helsinki, and informed consent was obtained from all participants.

**Table 1:** The demographic information of the participants; the participants are divided into five groups based on their age.

Age groups		Group 1 (20–30 years)	Group 2 (31–40 years)	Group 3 (41–50 years)	Group 4 (51–60 years)	Group 5 (61–70 years)
Gender	Female	30	31	31	27	14
	Male	29	31	24	23	12
YoE mean (±std)		16.89 (±2.17)	16.81 (±3.92)	14.62 (±3.27)	13.97 (±4.06)	13.53 (±3.31)

The number of participants in each group are provided. YoE, Years of Education, provided as mean (±std); std., standard deviation.

### 2.2 Cognitive tests

In this research, the data of two widely used measures, the N-back task and the forward and backward digit span tests, were used to investigate the working memory.

#### 2.2.1 N-back task

One of the most popular tasks in cognitive neuroscience studies to measure working memory performance is the n-back task (Owen et al., 2005). This task typically involves presenting participants with a series of stimuli, and the objective is to determine whether each stimulus matches the one presented N items prior. The processing load increases with increasing value of N, which is indicated by a decrease in accuracy and an increase in reaction time (RT; Au et al., 2015). Its greater manipulation power and less complexity than other cognitive tasks are the reasons for the wide use of this task (Conway et al., 2003). It should be noted that in this research the one-back task was used. In the condition of a one-back test, the target is any letter that is identical to the letter immediately preceding it. So in the letter sequence “N-R-Y-C…,” the participant should respond “match” if the 5th letter in the sequence were a “C” because it matches one previous letter. This task was presented on a laptop connected to a button box on which participants made their responses. All participants used their index fingers to press a specified button. Stimuli were randomly presented at a fixed central location on the computer screen. Also, Stimuli were randomly presented at a fixed central location on the computer screen for 500 ms with an inter-stimulus interval of 2,500 ms. Prior to the start of the actual task, participants were trained until they demonstrated that they understood the task and their performance stabilized. The trials used for practice were not used in the main task. The time required for this test was 10 min. At the end, the accuracy percentage score for each person’s performance in this test was obtained. Increasing the difficulty of the N-back task might have been cognitively difficult for the older participants. Using a challenging task could lead to fatigue and frustration. Also, By using the 1-back task, we aimed to establish a baseline level of performance before progressing to more difficult tasks. This can provide valuable information about participants’ cognitive abilities and help determine appropriate difficulty levels for future studies.

#### 2.2.2 Digit span test

A prevalent measure for the assessment of verbal working memory is the digit span test (Ostrosky-Solís and Lozano, 2006). Digit Span test requires subjects to repeat series of digits of increasing length. This test can be used in two formats, forward digit span test (FDST) and backward digit span test (BDST). In the digit span test, first a series of numbers are presented audibly and the examiner asks the subject to repeat those digits, In the FDST, the examiner asks the subject to repeat the numbers in the same order they were read aloud. In the BDST, the examiner asks the subject to repeat the numbers in the reverse order of the numbers presented by the examiner. The presentation rate of digit spacing and pitch should be consistent with standard procedures. The presentation of digit spacing was 1 s apart. Constant pitch should be used to pronounce all digits, meaning that we did not have to change the pitch when pronouncing each digit in a sequence. Varying voice pitch may facilitate the use of a chunking strategy, which may lead to overestimation of ability. Also, repetition was not allowed in the digit span. If the subject wanted us to repeat the sequence, it should be said: “I can only say the numbers once, just make your best guess.” The presentation starts with two digits in a series and the difficulty level of the test increases with the presentation of up to 9 digits in a series. It should be noted that the score in the two-digit series is not considered. In the present study, each series is repeated three times and one score is given for each correct answer. For this purpose, first, each series was repeated three times and then the difficulty of the test was increased. The series of digits in the three series were always different. It should be mentioned that a percentage correct for each trial was considered for the analyses. Due to the fact that the score is calculated from 3-digit to 9-digit series, the maximum score of the participants in each forward and backward format is 21.

### 2.3 MRI scanning

The MRI machine used in this study was a Siemens 3.0 Tesla scanner (Prisma, 2016), devoted to research, at the Iranian National Brain Mapping Lab.^1^ We used a 64-channel head coil in our study. The MRI protocols were selected to match the international projects, such as the UK Biobank or the ENIGMA consortium. The MRI protocols were as follows.

Resting-state fMRI: Total time = 6 min; TR = 2,500 ms; TE = 30 ms; flip angle = 90 degrees; voxel size = 3.0 × 3.0 × 3.0 mm; #slices = 40; matrix size = 64 × 64 × 40; Time-points = 144.

Structural T1-weighted MPRAGE: Total time = 4:12 min; TR = 1800 ms; TE = 3.53 ms; flip angle = 7 degrees; voxel size = 1.0 × 1.0 × 1.0 mm; #slices = 160; matrix size = 256 × 256 × 160.

All MRI data were visually checked for good quality, based on previous methods (Sisakhti et al., 2021, 2022). This step included image information such as matrix and voxel sizes, the number of time-points (for resting-state fMRI), and checking the images to be right-to-left oriented. Besides, the visual check was performed to spot possible macroscopic artifacts and vibration/motion evidence in images, and to check head tilt and head positioning, signal loss, ghosting, or other possible artifacts in the data.

### 2.4 Data analysis

#### 2.4.1 Resting fMRI data analysis

Details of our data analysis were published previously (Alemi et al., 2018). In summary, first, the fMRI data underwent seven steps of preprocessing, including slice timing correction, realignment, co-registration, normalization, smoothing, segmentation, and motion correction. Slice timing section was performed using the following settings: number of slice = 43; TR = 2,500 ms; TA = 0.9768 (1–1/43). For realignment, the settings were: quality = 0.9; separation = 4; smoothing = 5; and interpolation = 5. To perform the co-registration step, we chose the T1 image as the reference image, and all volumes of the resting state images were chosen as the source images. In normalization, for the image to align we selected the T1 image, and for image to write, we selected all volumes of the resting state images that were extracted from the last preprocessing step (co-registration). The setting of smoothing was: FWHM = 6; data type = same; implicit masking = none. For motion correction, the MCFLIRT toolbox, utilized in FSL, was used, and the criteria for including the data with an acceptable motion was the absolute displacement (rotation and translation) being less than 2.0 mm.

Also, one preprocessing step was performed on the structural T1-weighted images, which included removing the skull and non-brain tissues from the T1-weighted brain images. FSL (FMRIB Software Library v6.0 Created by the Analysis Group, FMRIB, Oxford, United Kingdom) has a tool for this called BET (Brain Extraction Tool), and we used it with these settings: fractional intensity threshold = 0.35; bias field and neck cleanup.

We used the MELODIC toolbox (Multivariate Exploratory Linear Optimized Decomposition into Independent Components), from the FSL software package (FMRIB Software Library v6.0 Created by the Analysis Group, FMRIB, Oxford, United Kingdom), in order to identify the brain activation maps during the resting state; these brain activations are referred to as independent components in the spatial ICA algorithm performed in Melodic, FSL. Independent Component Analysis is used to decompose a single or multiple 4D data sets into different spatial and temporal components.

The preprocessed data were imported into MELODIC (group ICA analysis, temporal concatenation approach), in order to pick out different activation and artifactual components without any explicit time series model being specified. The settings of the MELODIC analysis included: number of inputs = 252; slice timing correction = interleaved; motion correction = MCFLIRT; spatial smoothing FWHM = 5 mm; activate intensity normalization; multi session temporal concatenation mode of analysis; and Threshold IC maps = 0.9. Running the ICA analysis on these 252 resting-state fMRI data, based on the above settings and by the temporal concatenation approach, resulted in 109 independent components for all the 252 fMRI data. Each independent component represents a particular pattern of brain activation or artifact, observed in common in all the 252 data and during the resting state.

These 109 components included the maps relevant to the task-evoked activations, relevant to the intrinsic activities of the individuals during the resting-state, as well as the maps relevant to the artifacts or other confounding factors. Based on our hypothesis in this work, and based on previous works (Tang et al., 2017), we identified the following functional networks among our results, and the other steps of the analysis were merely performed on these networks: anterior and posterior default mode network (Ant-DMN & post-DMN), right and left executive control network (R-ECN & L-ECN), and salience network (SN), resulting in five networks. Identification of these networks were based on visual inspection of the output functional networks.

Dual regression is a tool that we can use as part of a group-level resting state analysis to identify the subject-specific contributions to the group level Independent Component Analsis (ICA). The output of dual regression is a set of subject-specific spatial maps and time courses for each group level component (spatial map) that can then be compared across subjects/groups. All steps of dual regression were applied in FSL software. We applied dual regression on the outputs of the MELODIC ICA by a very simple code in the virtual machine of Linux in the WINDOWS environment. The Dual Regression coding (example code: dual_regression group_IC_maps des_norm design.mat design.con n_perm output_directory input.filelist) was applied on the outputs of the MELODIC ICA step, where the inputs were the 109 components estimated for all the 252 participants. The outputs of this step were used to quantify the strength of the activation of each of the five networks in the 252 participants of the study. The strength was defined as the average z-value of the activated voxels (z-value>2.3) in the network.

#### 2.4.2 Volumetric data analysis

Details of our volumetric analysis methods were published previously (Batouli et al., 2014a; Batouli and Saba, 2021; Keihani et al., 2017). As a summary, initially the quality of the T1-weighted scans was visually checked for a correct orientation and matrix and voxel sizes. The visual check was also performed to spot possible macroscopic artifacts and vibration/motion evidence in images, for a proper signal-to-noise ratio, and to check head tilt and head positioning, signal loss, ghosting, or other possible artifacts in the data.

Next, voxel-based morphometry (VBM) analysis (Ashburner and Friston, 2000) was performed as follows. The T1- weighted scans were segmented into gray matter volume (GMV), white matter (WM), and cerebrospinal fluid (CSF), using the Segment toolbox, SPM12, which created the Native Space plus Diffeomorphic Anatomical Registration Through Exponentiated Lie Algebra (DARTEL) imported outputs (Ashburner, 2010); using the default settings of the “Run DARTEL: create template” toolbox, the accuracy of inter-subject alignment was improved by iteratively averaging the DARTEL-imported data of the GMV and WM tissue types to generate population-specific templates; and after generation of the templates, all the GMV and WM images were normalized to the Montreal Neurological Institute (MNI) standard space, using the “Normalize to MNI space” toolbox.

The aim of this analysis was to estimate the volume of several brain regions, and as a result, two brain atlases were used in this section, including the Desikan-Killiany Atlas (Alexander Loh et al., 2019) and the Aseg Atlas (Fischl et al., 2002; Sederevičius et al., 2021). The atlases provided the ROIs of brain areas, and then the volume of an ROI was calculated by adding the probability estimates of the GMV and WM maps, and then multiplying the resulted value to 3.375 mm3 (the volume of one voxel), using a code written in MATLAB. In this section, the volume of 85 brain regions were estimated for all the 252 participants. Generally, 85 gray matter volumes and 5 resting-state networks were included in the statistical analysis of the study.

### 2.5 Statistical analysis

This study aimed to examine the mediating effects of the brain structures and resting-state brain networks on the relationship between age and working memory. The preliminary analysis was done using SPSS version 26 and the mediation analysis was done using AMOS version 24. The steps of statistical analysis were as follows: first, the mean and standard deviation of the study variables (including independent, and dependent variables) were estimated. Skewness and kurtosis were also reported, which indicated the normal distribution of the data. In the next step, correlation analysis was performed between all study variables. It should be noted that age was included as an independent variable, brain structures and resting-state brain networks as mediators, and working memory as the dependent variable. The correlation analyses were first performed between age and each score in the cognitive tasks, then the correlation between age and each of the brain structures and resting-state brain networks was performed; and then, the correlation between brain imaging measures and each score in cognitive tasks was calculated. It should be mentioned that to reduce the risk of false positive discoveries due to multiple comparisons effect, the study utilized the Bonferroni approach as a subset of the Family-Wise Error Rate (FWER) multiple comparison corrections, setting the adjusted significance level at 0.00052. This level of *p*-value was applied to all our analyses, and therefore the reported results are FWER-corrected.

Finally, the mediation effects of the brain structures and resting-state brain networks on the relationship between age and each score in cognitive tasks were investigated. To identify the direct and indirect effects of age on working memory, the correlation between the paths of the hypothetical model was calculated and the non-significant paths were removed step by step. It is noteworthy that years of education were included as the control variable in the path analysis. Controlling for years of education helps us provide more accurate and meaningful insights into the relationships between variables and ensures differences in outcomes are not simply due to variations in years of education. Finally, the acceptable empirical model was examined.

## 3 Results

### 3.1 Preliminary analyses

#### 3.1.1 Descriptive statistics

First, the mean, standard deviation (SD), and range of the age, n-back test, forward digit span task and backward digit span task tests were computed. The mean accuracy in the one-back task for all the participants was 90.98 ± 12.79%. The mean total forward and backward digit span task for all the participants were 8.20 ± 1.94% and 6.21 ± 1.99%, respectively. The ranges for the kurtosis and skewness of each data ranged from −1 to +1, indicating that age, n-back test, FDST and BDST were normally distributed (Hair et al., 2022). The results by age groups are given in Table 2.

**Table 2:** The descriptive statistics of the study variables.

Measures	Group 1 (20–30 years)		Group 2 (31–40 years)		Group 3 (41–50 years)		Group 4 (51–60 years)		Group 5 (61–70 years)		Whole Sample	
	Mean (SD)	Range	Mean (SD)	Range	Mean (SD)	Range	Mean (SD)	Range	Mean (SD)	Range	Skewness	Kurtosis
Age	25.15 (2.88)	20–30	35.32 (3.11)	31–40	45.69 (2.99)	41–50	54.80 (3.01)	51–60	65. 46 (3.10)	61–70	−0.01	−0.21
N-back accuracy %	92.74 (13.60)	2.50–100	89.56 (14.81)	21.67–100	92.46 (7.42)	59.17–100	90.51 (13.69)	9.17–99.17	88.14 (12.85)	47.50–98.33	0.003	−0.21
Tot FDST	9.22 (1.75)	6–14	8.92 (1.97)	5–14	7.56 (1.77)	4–12	7.36 (1.67)	4–12	7.15 (1.34)	5–9	0.38	0.04
Tot BDST	7.39 (2.04)	3–13	6.82 (1.98)	3–12	5.51 (1.46)	2–11	5.34 (1.71)	1–10	5.27 (1.56)	3–9	0.54	0.50

The participants are divided based on their age, and the mean and standard deviation of the cognitive measures for the one-back task, and the forward and backward digit span tasks, are provided. SD, Standard Deviation; FDST, Forward Digit Span Task; BDST, Backward Digit Span Task.

Subsequently, the intervening role of years of education and gender on variables was examined. The Pearson correlation analysis revealed that there was a significant correlation between years of education and the score of the n-back test, forward and backward DST (*r* = 0.30, *p* < 0.01; *r* = 0.36, *p* < 0.01; *r* = 0.44, *p* < 0.01, respectively). The correlation coefficients are presented in Table 3. Also, the T-test analysis showed no significant differences between males and females in the n-back test [t (df = 250) = −1.03, *p* > 0.05], FDST [t (df = 250) =0.97, *p* > 0.05], and BDST [t (df = 250) = −0.72, *p* > 0.05]. The analyses indicated that years of education could play the role of an intervening variable in working memory, but not gender.

**Table 3:** The correlation coefficients between age and education and cognitive tests, and the *t*-test results for gender differences in the study variable; YoE, Years of education; **p-value < 0.01.

	Correlation with age	Correlation with YoE	M vs. F (*p*-value)
Age	–	−0.37**	0.40
N-back accuracy %	−0.06	0.30**	0.30
Total FDST	−0.41**	0.36**	0.33
Total BDST	−0.42**	0.44**	0.46

#### 3.1.2 Age associations

The results of the Pearson correlation analysis indicated that there was no significant correlation between age and the score of the n-back test (*r* = 0.06, *p* > 0.05). However, both the FDST and BDST were significantly correlated with age (*r* = −0.41, *p* < 0.01; *r* = −0.42, *p* < 0.01; respectively), suggesting that an increased age was associated with a poorer working memory performance.

According to the VBM analysis, 85 cortical and subcortical gray matter volumes were obtained in this study. The Pearson correlation analysis revealed a significant and negative correlation between age and 80 of those volumes, suggesting a decreased GMV with increasing age.

In our RSN analysis, five resting-state networks, namely the anterior and posterior default mode network (DMN), right and left executive control network (ECN), and salience network (SN) were selected. The association of the level of the activity of these networks with age showed that the anterior default mode network, left executive control network, and salience network exhibited a negative correlation with age (*r* = −0.26, *p* < 0.01; *r* = −0.23, *p* < 0.01; *r* = −0.22, *p* < 0.01; respectively). To address the issue of false-discovery bias when conducting multiple comparisons, the Bonferroni correction was employed, which adjusted the significance level to 0.00052.

#### 3.1.3 The neural correlates of working memory

To investigate the neural correlates underlying working memory, correlation analysis was performed between GMVs and the RSNs with the working memory measures. The correlation analysis showed that the GMV in 56 and 31 brain structures significantly correlated with the FDST and BDST, respectively. On the other hand, no significant correlation was observed with the one-back test results. Similarly, the activity levels of the RSNs did not show a significant correlation with the WM measures. The results of this section are provided in Table 4.

**Table 4:** The coefficients of the correlation between the brain volumes and age, FDST and BDST measures.

Brain Structure	Age correlation		FDST correlation		BDST correlation	
	Left	Right	Left	Right	Left	Right
Inf. Occ.	−0.16^**^	−0.20^**^	0.12^*^	0.10	0.12	0.16^*^
Ant. Cingulate	−0.43^**^	−0.46^**^	0.26^**^	0.26^**^	0.19^**^	0.20^**^
Ant. Mid. Cingulate	−0.39^**^	−0.34^**^	0.29^**^	0.25^**^	0.21^**^	0.22^**^
Post. Mid. Cingulate	−0.41^**^	−0.33^**^	0.26^**^	0.28^**^	0.17^**^	0.20^**^
Mid. Frontal	−0.52^**^	−0.46^**^	0.33^**^	0.29^**^	0.29^**^	0.26^**^
Mid. Occipital	−0.38^**^	−0.35^**^	0.29^**^	0.21^**^	0.17^**^	0.20^**^
Sup. Occipital	−0.33^**^	−0.33^**^	0.21^**^	0.16^*^	0.18^**^	0.15^*^
Calcarine	−0.25^**^	−0.23^**^	0.16^*^	0.12^*^	0.14^*^	0.10
Ant. Occipital	−0.395^**^	−0.24^**^	0.21^**^	0.19^**^	0.17^**^	0.20^**^
Parieto-Occipital	−0.30^**^	−0.40^**^	0.19^**^	0.31^**^	0.23^**^	0.27^**^
Cuneus	−0.23^**^	−0.27^**^	0.13^*^	0.18^**^	0.12	0.14^*^
Entorhinal G.	−0.14^*^	−0.07	0.11	0.12^*^	0.15^*^	0.11
Fusiform	−0.32^**^	−0.34^**^	0.23^**^	0.20^**^	0.22^**^	0.18^**^
Inf. Parietal	−0.42^**^	−0.47^**^	0.26^**^	0.27^**^	0.15^*^	0.19^**^
Inf. Temporal	−0.41^**^	−0.46^**^	0.27^**^	0.26^**^	0.21^**^	0.24^**^
Lateral Occipital	−0.32^**^	−0.29^**^	0.23^**^	0.17^**^	0.17^**^	0.17^**^
Lateral Orbitofrontal	−0.55^**^	−0.56^**^	0.26^**^	0.26^**^	0.25^**^	0.22^**^
Lingual G.	−0.29^**^	−0.24^**^	0.17^**^	0.09	0.11	0.04
Medial Orbitofrontal	−0.46^**^	−0.46^**^	0.26^**^	0.30^**^	0.19^**^	0.20^**^
Mid. Temporal	−0.47^**^	−0.49^**^	0.27^**^	0.27^**^	0.23^**^	0.26^**^
Para-Hippocampal	−0.38^**^	−0.28^**^	0.22^**^	0.18^**^	0.27^**^	0.22^**^
Paracentral G.	−0.34^**^	−0.31^**^	0.25^**^	0.27^**^	0.16^*^	0.20^**^
Pars Opercularis	−0.42^**^	−0.39^**^	0.18^**^	0.22^**^	0.10	0.16^*^
Pars Orbitalis	−0.44^**^	−0.41^**^	0.19^**^	0.14^*^	0.15^*^	0.11
Pars Triangularis	−0.50^**^	−0.40^**^	0.20^**^	0.23^**^	0.19^**^	0.20^**^
Postcentral G.	−0.39^**^	−0.40^**^	0.23^**^	0.25^**^	0.15^*^	0.19^**^
Posterior Cingulate	−0.41^**^	−0.38^**^	0.24^**^	0.24^**^	0.18^**^	0.19^**^
Precentral G.	−0.45^**^	−0.41^**^	0.33^**^	0.22^**^	0.21^**^	0.14^*^
Precuneus	−0.47^**^	−0.51^**^	0.24^**^	0.27^**^	0.26^**^	0.26^**^
Sup. Frontal	−0.53^**^	−0.50^**^	0.26^**^	0.29^**^	0.23^**^	0.26^**^
Sup. Parietal	−0.43^**^	−0.39^**^	0.25^**^	0.25^**^	0.24^**^	0.21^**^
Sup. Temporal	−0.52^**^	−0.51^**^	0.27^**^	0.28^**^	0.29^**^	0.24^**^
Supramarginal G.	−0.44^**^	−0.45^**^	0.21^**^	0.25^**^	0.20^**^	0.18^**^
Insula	−0.43^**^	−0.43^**^	0.25^**^	0.22^**^	0.21^**^	0.19^**^
Cerebellum Cortex	−0.51^**^	−0.45^**^	0.23^**^	0.21^**^	0.27^**^	0.23^**^
Thalamus	−0.58^**^	−0.49^**^	0.41^**^	0.32^**^	0.29^**^	0.25^**^
Caudate	−0.48^**^	−0.41^**^	0.27^**^	0.24^**^	0.22^**^	0.18^**^
Putamen	−0.50^**^	−0.52^**^	0.32^**^	0.32^**^	0.25^**^	0.24^**^
Pallidum	−0.41^**^	−0.33^**^	0.23^**^	0.19^**^	0.20^**^	0.14^*^
Hippocampus	−0.40^**^	−0.38^**^	0.30^**^	0.29^**^	0.25^**^	0.23^**^
Amygdala	−0.44^**^	−0.34^**^	0.23^**^	0.27^**^	0.25^**^	0.21^**^
Accumbens Area	−0.38^**^	−0.46^**^	0.21^**^	0.24^**^	0.16^*^	0.25^**^
Brain Stem	−0.21^**^		0.15^*^		0.12	

*p-value<0.05; **p-value<0.001. Multiple comparison correction was implemented based on the Bonferroni (Family-Wise Error Rate) approach. Inf, inferior; Occ, occipital; Ant, anterior; Mid, middle; Sup, superior; G, gyrus.

### 3.2 The higher level (mediation) analysis

The mediation model was conducted to clarify the mediation role of the structural and functional brain measures in the relationship between age and working memory. Age was the independent variable of the path analysis, brain structure and resting state networks as the mediators, working memory as the dependent variable, and years of education as the control variable. Based on the preliminary analyses performed above, only the structural and functional brain measures which showed a significant correlation with age and the cognitive tests results were included in the model. Based on that, the measures of the one-back test were not involved in the model. The brain structures were divided into seven categories based on the parcellation of AAL-Atlas, including the frontal, parietal, temporal and occipital lobes, insula and cingulate, posterior fossa, and central structures (Rolls et al., 2020), and seven separate models were designed and tested.

**Figure 1:**
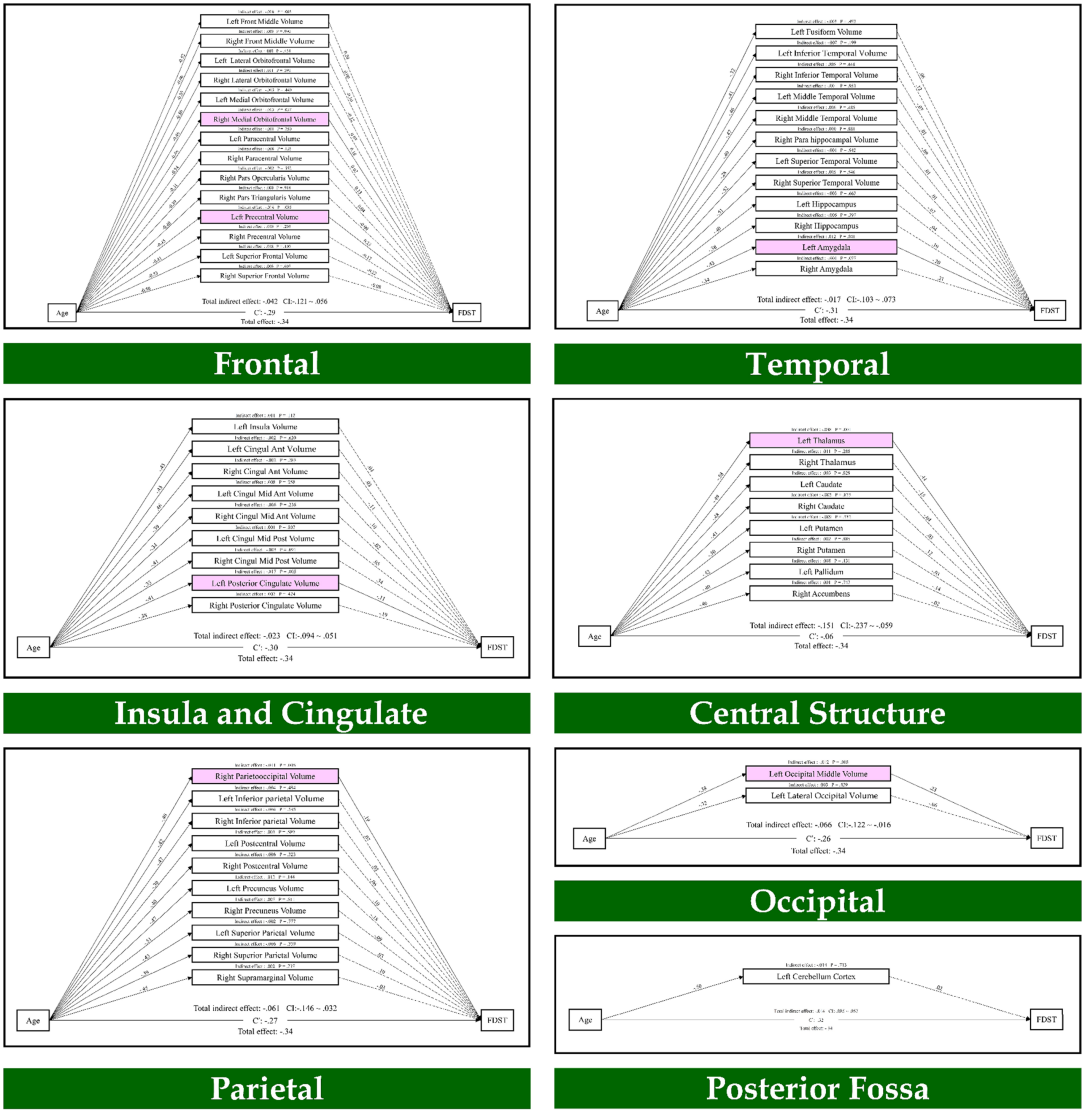
The mediation model (path analysis) between the brain volumes, age, and the forward digit span task scores. The solid lines indicate the statistically significant paths, and the dashed line indicates non-significant paths. The path values show the standardized beta weights and *p*-values. Pink rectangles indicate significant mediating variables. The confidence interval (CI) indicates 95% confidence interval for the indirect and total effects.

To elucidate the direct and indirect pathways, a path analysis was conducted to test whether structures and networks could mediate the relationship between age and working memory. As provided in Figure 1, which is relevant to the FDST, the results indicated that age through the frontal lobe (right medial orbitofrontal volume, left precentral volume; *β* = −0.01, *p* = 0.037; *β* = −0.01, *p* = 0.035), parietal lobe (right parietooccipital volume; *β* = −0.01, *p* = 0.005), temporal lobe (left amygdala; *β* = 0.01, *p* = 0.041), occipital lobe (left occipital middle volume; *β* = −0.01, *p* = 0.018), insula and cingulate (left posterior cingulate volume; *β* = −0.01, *p* = 0.005) and central structure (left thalamus; *β* = −0.03, *p* = 0.003) had an indirect effect on the FDST.

**Figure 2:**
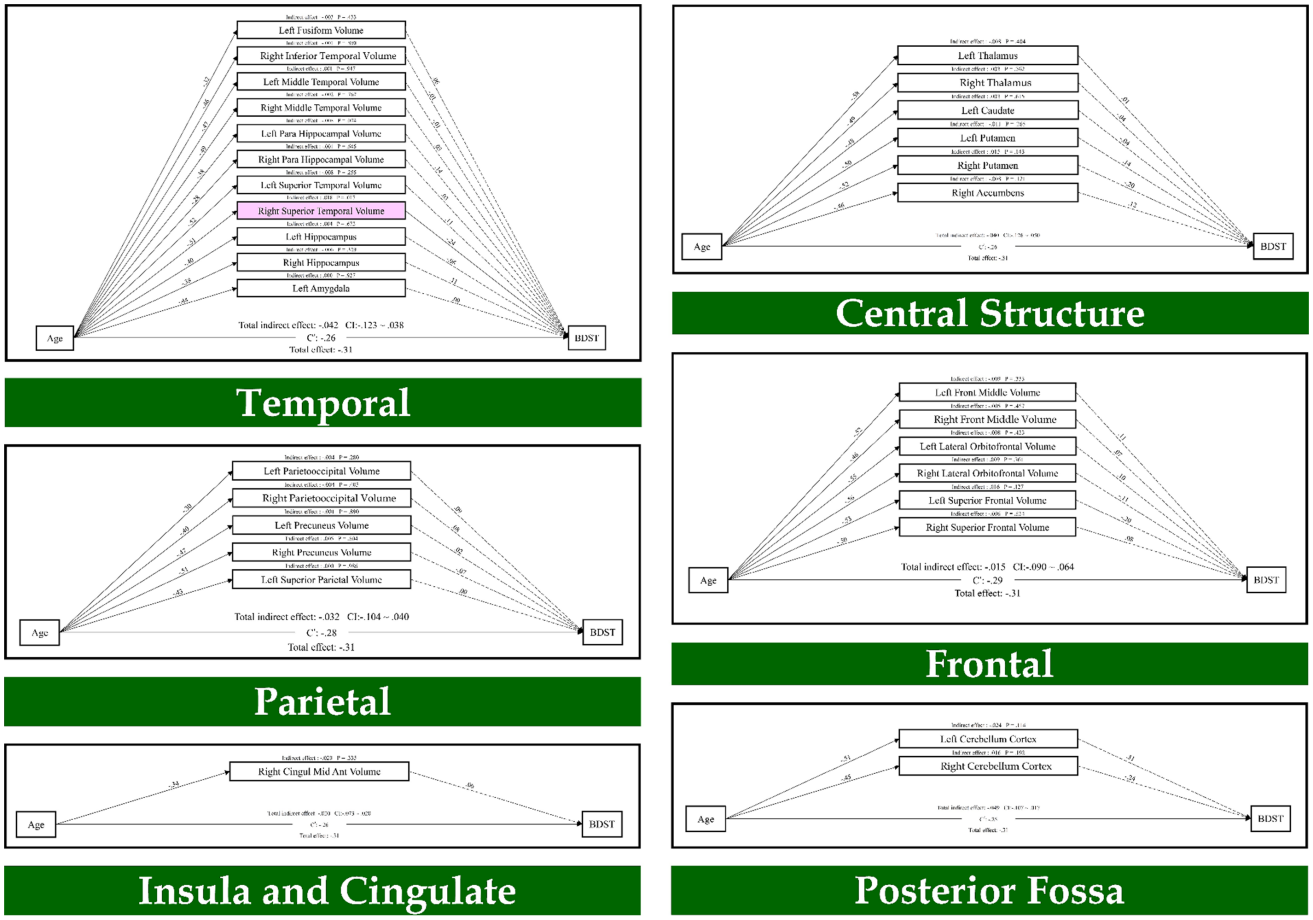
The mediation model (path analysis) between the brain volumes, age, and the backward digit span task scores. The solid lines indicate the statistically significant paths, and the dashed line indicates non-significant paths. The path values show the standardized beta weights and p-values. Pink rectangles indicate significant mediating variables. The confidence interval (CI) indicates 95% confidence interval for the indirect and total effects.

Also, the results of path analysis on the BDST, as provided in Figure 2, indicated that age through the temporal lobe (right superior temporal volume; *β* = 0.01, *p* = 0.017) had an indirect effect on the BDST. None of the resting state networks had an indirect effect on these tests. As a result, seven brain regions mediated the relationship of age with the FDST, and one brain structure mediated between age and the BDST.

## 4 Discussion

### 4.1 Summary of the results

The purpose of this study was to determine which brain structures or resting-state brain networks mediate age-related differences in working memory. First, the correlation analysis between age and working memory tasks showed that there is a significant negative correlation between age and FDST and BDST, but no significant correlation was observed between age and the n-back task. Then, according to the Pearson correlation analysis, correlations between age and 80 brain structures were significantly negative, suggesting lower GMVs with increasing age. In addition, there was a significant correlation between age and three networks (anterior default mode network, left frontal network, and salience network). In the next step, the correlation analysis showed that there is a relationship between 56 and 31 gray matter volumes with forward and backward digit span tasks, respectively. Furthermore, we found that none of the resting state networks had a significant correlation with working memory tasks.

Finally, the mediation analysis showed that the GMV in the right medial orbitofrontal, left precentral, right parietooccipital, left amygdala, left middle occipital, left posterior cingulate and left thalamus mediate the age-related differences in the forward digit span task. Furthermore, GMV in the right superior temporal mediated the age-related differences in the backward digit span task. Neither of the resting-state networks had an indirect effect on the forward and backward digit span tasks.

### 4.2 Working memory alters with age

Consistent with our hypothesis, age negatively correlated with the score in the forward and backward digit span tasks. The present results are consistent with prior literature relating age and working memory (Fabiani, 2012; Gajewski et al., 2018). Bosnes et al. (2022) found that working memory performance of healthy older adults is associated with the process of aging well. Similarly, in a study to investigate age-related changes in spatial working memory (Klencklen et al., 2017), two groups of adults aged 20–30 and 65–75 were compared. The results found that older adults performed less well on working memory tasks than younger adults.

Also, our research showed that there is no correlation between the age and performance of the participants in the one-back test. Consistent with the results, Cansino et al. (2013) investigated how the difficulty of a working memory task may affect age-related decline. They used the N-back task with two levels of difficulty in their research and showed that with increasing age, working memory accuracy decreased in 2-back tasks compared to one-back tasks. However, Mattay et al. (2006) showed that older subjects performed as well as younger subjects in the one-back task, and therefore it can be concluded that the effect of aging on working memory is dependent on the cognitive load of the task, and thus, when the cognitive demand of a task decreases, it is less affected with increasing age.

### 4.3 Age differences in brain measures

The present study showed that most volumes of gray matter in different brain structures have a negative correlation with aging. Consistent with our result, previous studies also have reported brain changes with age (Driscoll et al., 2009; Giorgio et al., 2010; Huang et al., 2015; Jockwitz et al., 2017; Varangis et al., 2019). For example, by examining the differences in brain volume among four groups of male and female older adults, Farokhian et al. (2017) found that GMV in the frontal, insular, and cingulate cortices was reduced in older adults compared to younger adults in both genders.

In the network-level analysis, we found that activity in three resting state networks, including the anterior default mode network, left frontal network, and salience network were correlated with aging. Consistent with our findings, studies have shown that increasing age is associated with a diminished activity in the ant-DMN and post-DMN networks (Damoiseaux et al., 2008; Jones et al., 2011; Koch et al., 2010; Sambataro et al., 2010). In addition, consistent with our result, studies have shown age-related changes and abnormalities in the frontal (Fujiyama et al., 2016) and salience (He et al., 2014) networks in normal aging. Also, a recent systematic review of large-scale resting-state networks in aging found that the brain of older adults is less efficient and modular at rest (Deery et al., 2023). Overall, the variations seen in brain gray matter volume during normal aging may explain the difference in cognitive performance among older individuals.

### 4.4 The mediation effect on brain measures

At the cerebral level, correlation analysis revealed that worse performance in working memory is associated with significantly smaller GMV in multiple brain structures, especially in the frontal, temporal, and parietal regions. These regions are known to be involved in the working memory performance (Emch et al., 2019; Nissim et al., 2017; Rottschy et al., 2012). Finally, the path analysis results showed that the frontal lobe (right medial orbitofrontal volume, left precentral volume), parietal lobe (right parietooccipital volume), temporal lobe (left amygdala), occipital lobe (left middle occipital volume), Insula and cingulate (left posterior cingulate volume) and central structure (left thalamus) mediate adult life span differences in the forward digit span task. Furthermore, we found that the temporal lobe (right superior temporal volume) mediates adult life span differences in the backward digit span task.

Consistent with our result, Nissim et al. (2017) in a research aimed at determining the neural correlates of reduced working memory performance in the frontal lobes, compared two groups of healthy elderly people with high and low working memory in terms of cortical thickness and cortical surface area. The results showed that the cortical surface area in the medial orbital frontal gyrus, inferior frontal gyrus, and superior frontal gyrus is significantly reduced in subjects with a low performance in working memory. In another study, Schulze et al. (2011) aimed to investigate working memory performance in healthy elderly using multimodal imaging techniques, comparing two groups of young adults (20–30 years of age) and the older (60+ years of age). The results showed a negative correlation between gray matter volume and reduced working memory performance in older adults. Also, the results showed that with increasing working memory load and increasing age, a significant increase in activation was observed in the left dorsal and ventral lateral prefrontal cortex. In another study, greater activity in the dorsolateral prefrontal cortex was observed in younger adults than in older adults during memory retrieval, which suggests that the dorsolateral prefrontal cortex mediates the age-related decline in working memory performance (Rypma and D’Esposito, 2000). Mattay et al. (2006) also showed lower performance with increased working memory load in older people compared to younger ones, and at the same time, showed less activity in the prefrontal regions. A recent study also reported that increasing age is associated with a linear decrease in the neural activation during spatial working memory performance in the related regions (Archer et al., 2018). In general, with a decrease in behavioral performance in the active memory, the neural activity in the related areas decrease, and with an increase in behavioral performance, the neural activity increases in the same areas in a corresponding manner.

Consistent with our results, studies have specifically reported age-related decreases in gray matter volume in the neocortex, including prefrontal, parietal, and temporal cortices (Giorgio et al., 2010), as well as deep structures such as the thalamus (Fama and Sullivan, 2015) and amygdala (Zanchi et al., 2017). The weakening of these regions, which can be the neural substrates of cognitive function (Smith et al., 2023), may be the basis of the observed differences in working memory. For example, MacHizawa et al. (2020) showed in a study that a greater volume of gray matter in the left lateral occipital region is associated with better visual working memory performance. It has also been reported that memory performance in older adults is significantly related to the gray matter volume of the middle frontal gyrus and several regions of the temporal lobe (Van Petten et al., 2004). Inconsistent with our results, Piras et al. (2010) reported that there is no significant relationship between thalamic gray matter volume and WM performance. In contrast, Van De Mortel et al. (2021) reported a reduction in thalamus volume as one of the earliest signs of cognitive decline in Mild cognitive impairment. It should be noted that our study aimed to identify for the first time the mediating role of gray matter volume in certain areas of the brain in the relationship between age and working memory.

It is important to highlight that in our work differential brain structures mediate the relationship between age and the forward versus backward digit span task. One explanation for the differential brain structures between forward and backward digit span is that these two tasks require different cognitive demands. Overall, the backward digit span involves more spatial processing and higher cognitive control compared to the forward digit span. For example, the backward digit span is associated with greater activation of the left occipital visual area, left prefrontal cortex, right dorsolateral prefrontal cortex, frontal eye field, frontal operculum cortex, anterior insular cortex, and dorsal anterior cingulate cortex (Donolato et al., 2017). In our work, the right superior temporal volume mediates the relationship between aging and the backward digit span task. One possible interpretation for our finding is the involvement of this region in processing both object- and space-related information. Therefore, its role in the backward digit span task is to be expected.

## 5 Limitations

The present study has a number of limitations. First, two tasks (n-back task and digit span test) were adopted to evaluate working memory. It is noteworthy that the selection of different tasks may produce different results. To measure working memory more accurately, it is suggested to use various working memory tasks both in terms of difficulty level and type (visual and verbal) in future studies. Moreover, in the present study, resting-state networks and voxel-based morphometry were used to investigate the neural correlates related to working memory. It is suggested that multimodal brain imaging measures can be used in future studies to obtain a more accurate measure of neural correlates related to working memory. Thirdly, considering that the cross-sectional study does not provide any information about the changes in gray matter volume and the decrease of working memory over time, it is suggested that future studies use a longitudinal approach to investigate the extent of GMV changes corresponding to working memory. Also, we tested the mediation role of the variables, although selecting an approach for actually testing the causality between the measures would be preferable.

In summary, we successfully demonstrated that GMV in multiple brain structures mediate age-related differences in working memory performance. Our findings go beyond previous research on age-related WM decline. WM as an executive function is crucial for learning, working, and managing daily life. Our results are consistent with the reports regarding the decrease in GMV with age and its effect on cognitive performance such as working memory. In general, our results support the view that some specific brain structures can be the basis of specific cognitive functions. We conclude that identifying brain structures mediating the relationship between age and working memory may provide an opportunity for early detection of individuals at risk for age-related memory decline, as well as an opportunity to design strategies aimed at reducing or preventing age-related memory decline.

## Data Availability

The original contributions presented in the study are included in the article/supplementary material, further inquiries can be directed to the corresponding author.

## Footnotes

^1^ www.nbml.ir
